# Editorial: B cell heterogeneity in the single-cell era

**DOI:** 10.3389/fimmu.2022.1112849

**Published:** 2022-12-19

**Authors:** Hector Cordero, Pedro Perdiguero, Emmanuel Zorn

**Affiliations:** ^1^ Columbia Center for Translational Immunology, Department of Medicine, Columbia University Irving Medical Center, New York, NY, United States; ^2^ Department of Genetics, Physiology and Microbiology, Faculty of Biological Sciences, Complutense University of Madrid (UCM), Madrid, Spain

**Keywords:** B cells, plasma cells, next generation sequencing - NGS, single-cell RNA sequencing (scRNAseq), immunity, thymus, spleen, blood

Advances in next-generation sequencing technologies have recently allowed interrogation of the immune system at the level of individual cells. Single-cell RNA sequencing is now widely used in B cell studies seeking to solve previously underrecognized cellular heterogeneity, define key processes in cell differentiation, understand the gene regulatory networks and predict their functions.

Our Research Topic has received the work of 31 contributors that aimed to elucidate the phenotype and function of B cells using different models. In the cancer field, Janjic et al. identified six different clusters of B cells in patients with head and neck squamous cell carcinoma (HNSCC), including germinal center, memory, memory-like B, switched memory, naïve and plasma cells. Germinal center B cells expressed high levels of transmembrane tumor necrosis factor (TNF), lymphotoxin alpha and beta, and TNF-related apoptosis-inducing ligand (TRAIL), suggesting that tissue-resident B cell subsets, and particularly germinal center B cells, may have higher innate cytotoxic potential than those in circulation. This study revealed that plasma cells, which are primarily found in the tumor and not blood, express the highest levels of granzyme B (GZMB), suggesting a different mechanism of action in the tumor microenvironment. Finally, the authors show that resting human B cells isolated from peripheral blood induce apoptosis of, and efficiently kill a large variety of leukemia and solid tumor cell types.

In humans, Zhao et al. studied hypersplenism (HS) at single-cell resolution, a symptom characterized by having an abnormally large and active spleen due to a variety of diseases, including viral infections and hepatocellular carcinoma. By taking this uncommon approach, authors elucidated cell types and genes involved in human HS. The majority of splenic cells were T and B cells (62% and 27%, respectively). Although this study was limited by the number of samples, authors found up to 9 clusters of B cells with the expansion of HLA-DRB5+ B-cells in one patient with HS caused by hypertension and hepatitis C viral infection. Authors also revealed that CCL5+ B-cells were exclusively present in the healthy control subject, supporting a deteriorated role in the splenic chemotaxis in HS condition, while a novel B cell cluster characterized by the expression of SNAI1+ was found in an HS patient caused by hepatitis C viral infection. In addition, a cluster of B cells that expressed SESN1+ were expanded in another HS patient caused by hepatocellular carcinoma subject. Among those highly expressed genes screened in B cells, only POLR2L was highly expressed in the healthy subject. Finally, authors also carried out a developmental trajectory analysis inferring that globally HS patients were similar to the control subject, but the T-cells and B-cells were in a state of incomplete development in HS condition patients. Overall, this study highlights the complexity of hypersplenism, giving some new insights into the cell types and phenotype of this symptom at single-cell resolution.

Kibler et al. identified a major splenic marginal zone B-cell subpopulation characterized by the lack of CD27 that is present in children and decreases with age. Authors stated that this B cell population is an antigen-experienced population carrying low levels of somatic hypermutations very early in life.

SoRelle et al. used immortalized lymphoblastoid cell lines (LCLs) with Epstein-Barr virus (EBV) to study the germinal center (GC) dynamic of B cells *in vitro*. These authors used ICAM1 as a key marker for defining the activated B cell phenotype, and CD27 for defining the memory B cell phenotype, and used a combination of these markers for setting three identified subpopulations (ICAM-1^hi^/CD27^lo^, ICAM-1^lo^/CD27^hi^, and ICAM-1^lo^/CD27^lo^). At single-cell detail, the activated subpopulation (ICAM-1^hi^/CD27^lo^) corresponded to light zone-like phenotype in LCLs, ICAM-1^lo^/CD27^hi^ corresponded to memory B cell phenotype. As transitional stages, authors identified a cluster of actively cycling cells expressing MKI67 and CDK1, consistent with the GC dark zone state. In addition to cycling cells, authors described another cluster of a pre-GC activated B cell precursor to early memory B cells (AP-eMBCs) that expressed CCR6 and CD22. Furthermore, the phenotype dynamics observed led the authors to propose a model in which EBV infection perpetuates a core loop of host B cell entry, engagement, exit, and re-entry into a GC-like reaction *in vitro*. In addition to canonical plasma cell markers, EBV+ plasma cells also expressed high levels of interferon response genes including, IFI35, IFITM1, OAS1, MX1, and IFNG-AS1, which enhances IFNG production, suggesting an important role of plasma cells on EBV-mediated infections.

In mice, Hagen et al. studied the role of a locus that encodes three mature miRNAs, which generally regulate mRNA stability and translation. Particularly, authors selectively inactivated the miR-142 locus in B6 mouse B cells. The ablation of miR-142 locus strongly diminished the mature B cell compartment in lymph nodes, suggesting a clear role in the pre-B cell formation during development. Notably, this locus also seems to be key for the establishment of the migratory properties, and modulation of the behavior required for naïve follicular B cell patrolling activity in the organism.

Finally, Gao and Cockburn reviewed the development and functions of CD11c^+^ atypical B cells (ABCs). Authors highlighted that ABCs were detected by unsupervised clustering regardless of the tissue studied, frequently marked by genes such as FCRL4+ FCRL5+ CD11c+ T-bet+ ZEB2+ and FGR+, suggesting that ABCs identified in different tissues and diseases shared a conserved transcriptome profile. T-bet and ZEB2 are often considered key transcription factors for ABC formation. The role of ABC as plasma cell precursors and as antigen-presenting cells (APC) is further discussed. Authors also remarked that through single-cell trajectory analysis ABCs form a separate developmental branch distinct from well-established B cell lineages such as activated B cells and memory B cells.

As editors of this Research Topic, it was our pleasure to review a wide range of fascinating articles and reviews. All these contributions to the B cell field allow the scientific community to make progress in the application of therapeutic strategies helping to prevent disease progression and building a robust map of the B cell diversity as key components of the adaptive immune system.

## Author contributions

HC conceived and wrote the editorial. EZ and PP have reviewed and approved the final version.

